# Case Report: Extensive Gangrene: A Rare Presentation of Multisystem Inflammatory Syndrome in Children

**DOI:** 10.4269/ajtmh.22-0252

**Published:** 2022-11-14

**Authors:** Kiran Kumar Banothu, Parag Shankarrao Dekate, Priyanka Gupta, Avinash Reddy, Satish Rao Immaneni

**Affiliations:** ^1^ Department of Pediatrics, Krishna Institute of Medical Sciences, Kondapur, Hyderabad, India;; ^2^ Department of Pathology, Krishna Institute of Medical Sciences, Secunderabad, India

## Abstract

A wide spectrum of cutaneous manifestations are reported in multisystem inflammatory syndrome in children (MIS-C). However, gangrenous changes are less frequently reported. A 3-year old boy, with a known case of unoperated tetralogy of Fallot, presented with a short history of fever, rash, and melena. The rash was black and diffuse, with a rapid progression. The patient was stable hemodynamically at admission, with pallor, grade II clubbing, edema, and oral ulcers. Inflammatory markers were raised. He developed gangrenous changes over the ears and acral areas. He had very high levels of antibodies to severe acute respiratory syndrome coronavirus2 (SARS-CoV-2) infection and was diagnosed as having MIS-C. Skin biopsy revealed near total epidermal necrosis with dermal vascular thrombi and negative immunofluorescence. Skin biopsy was positive for IgG antibodies to SARS-CoV-2. He was treated with antibiotics, immunomodulation with steroids, intravenous immunoglobulin, and plasmapheresis. He had features of both micro- and macroangiopathy. Gradually the child improved, with residual deformity.

## INTRODUCTION

Coronavirus disease 2019 (COVID-19) infection causes mild disease in a majority of children.^1^ Skin manifestations have been reported in approximately 0.2% to 20.4% patients with COVID-19 infection.^2^ Multisystem inflammatory syndrome (MIS) is typically seen in children (MIS-C) and presents with fever, rash, and gastrointestinal and cardiovascular involvement, and raised inflammatory markers. Cutaneous manifestations have been reported in up to 80% of children with MIS-C. A wide spectrum of skin manifestations are seen in MIS-C.^3^^,^^4^ Limited reports in MIS-C have reported gangrenous changes in children. Here, we present a child with unoperated tetralogy of Fallot (TOF) with extensive gangrene secondary to MIS-C who improved with appropriate care, with residual deformity.

## CASE PRESENTATION

A 3-year-old boy with a known case of unoperated TOF presented with a short history of fever for 5 days and rash for 3 days. The rash was black and started initially over the lower limbs, then progressed to involve the trunk, upper limbs, face, and ears during the following 3 days. He had two episodes of melena. He had no cough, breathing difficulty, loose stools, or vomiting.

**Figure 1.**
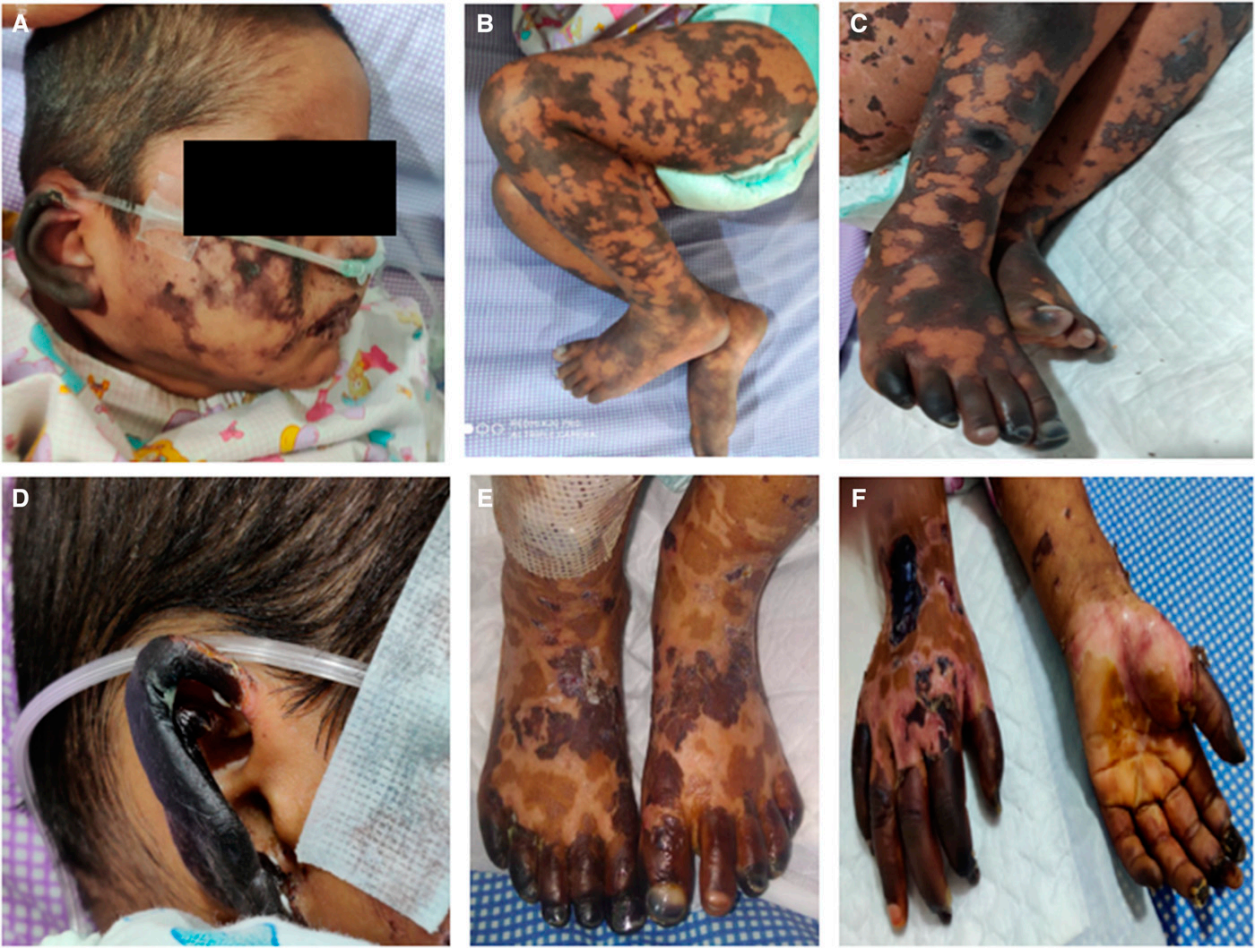
(**A** and **B**) Retiform purpura involving the face, ears, and lower limbs. (**C–F**) Gangrenous changes involving acral areas and ears.

On examination, the patient had tachycardia at 150 beats/minute, with a normal respiratory rate and blood pressure, and an oxygen saturation of 84% on room air. He had retiform purpura over the lower limbs, buttocks, trunk, upper limbs, and face (Figure 1A and B). The pinnae were black, with early gangrenous changes over the right ear. All peripheral pulses were well palpable. He had pallor, grade II clubbing, and generalized edema. Oral ulcers were present. There was no icterus, lymphadenopathy, or conjunctival redness. He had an ejection systolic murmur on the left sternal border, and the liver was 2 cm below the right coastal margin with a span of 8 cm. Respiratory and neurological examination was normal. Initial possibilities of meningococcemia, scrub typhus, purpura fulminans, or infection-induced small-vessel vasculitis were considered.

Laboratory investigations revealed hemoglobin, 10.6 g/dL; total leukocyte count, 34,600/mm^3^ (N, neutrophils 60%; L, lymphocytes 30%; M, monocytes 8%; and E, eosinophils 2%); platelet count, 91,000/mm^3^; urea, 79 mg/dL; and creatinine, 0.5 mg/dL. He had mild transaminitis (aspartate aminotransferase, 81 U/L; alanine aminotransferase, 36 U/L) and hypoalbuminemia (2.6 g/dL). Inflammatory markers were elevated (C-reactive protein, 175 mg/dL; erythrocyte sedimentation rate, 40 mm/hour). A workup for rickettsial disease using the Weil Felix test, meningococcus, and dengue were negative. Blood culture at admission was sterile. A COVID rapid antigen test was negative. A chest X-ray showed normal lung fields. Ultrasound revealed right-side minimal pleural effusion and mild ascites. The child was started on ceftriaxone, vancomycin, and doxycycline.

**Figure 2.**
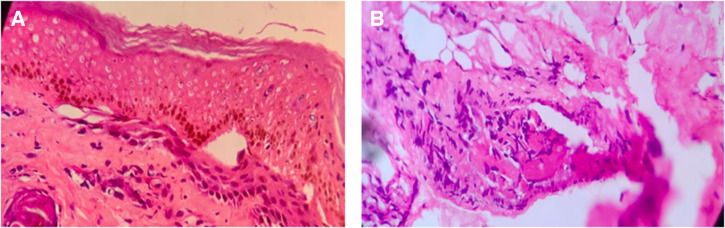
Skin biopsy showing epidermal necrosis and thrombosis of the blood vessels of the dermis.

After ruling out infectious causes, the possibility of MIS-C was considered. Antibodies to severe acute respiratory syndrome coronavirus2 (SARS-CoV-2) were highly elevated (IgG, 2,500 U/mL; cutoff, < 0.8 U/mL). Ferritin (866 ng/mL) and D-dimer (2,232 ng/mL) were elevated as well. Echocardiography showed TOF with severe right ventricular outflow track obstruction, borderline pulmonary artery, normal pulmonary valve annulus, normal cardiac contractibility, and normal coronaries. Skin biopsy revealed near-total epidermal necrosis, with dermal vascular thrombi and negative immunofluorescence (Figure 2A and Figure B). Antinuclear antibodies and antineutrophil cytoplasmic antibodies were negative.

**Figure 3.**
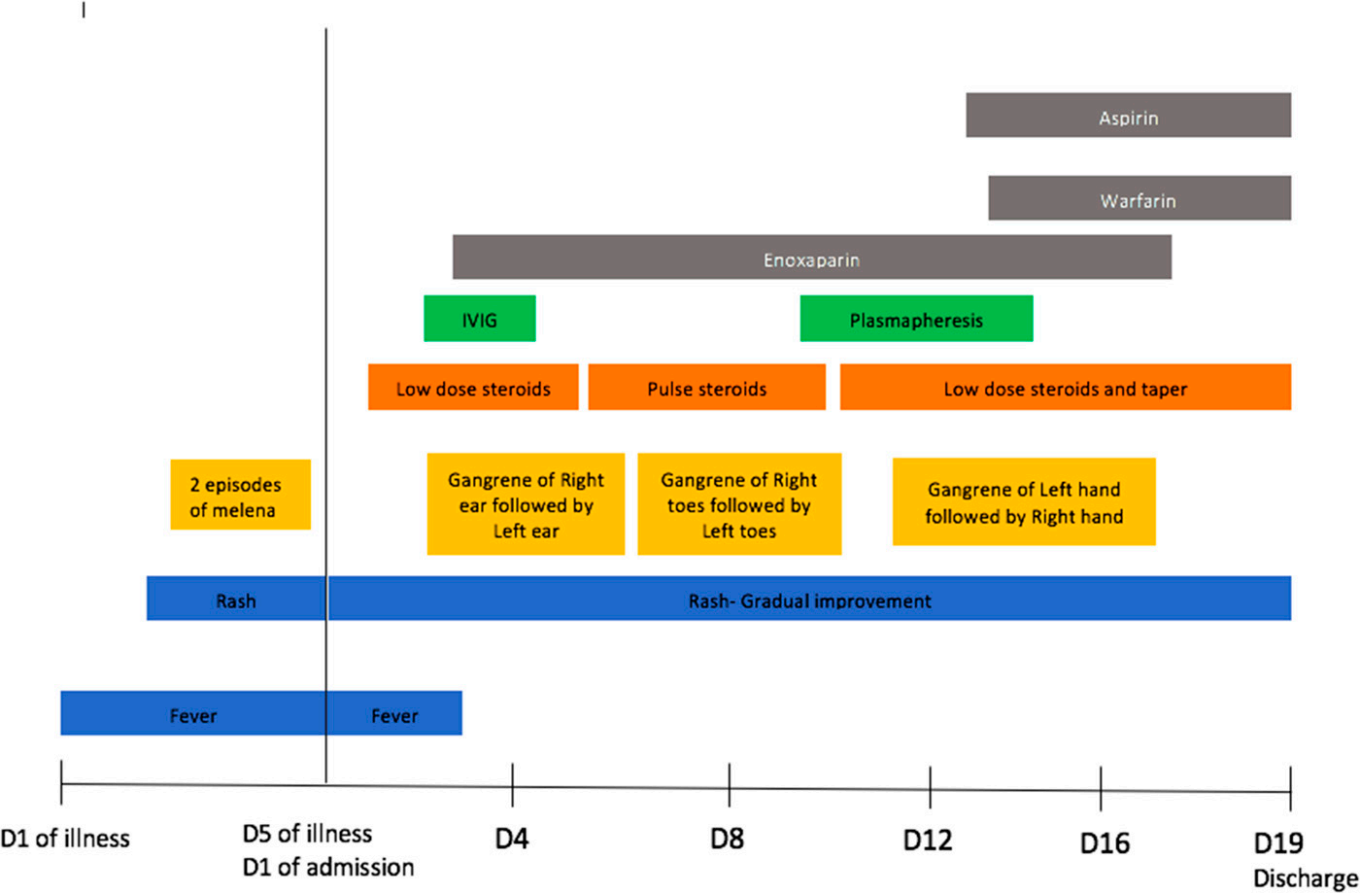
The clinical course of and interventions for the patient. D = day; IVIG = intravenous immunoglobulin.

The child was given intravenous immunoglobulin (IVIG) at 2 g/kg. He also received steroids at 20 mg/kg for 5 days and then a low-dose steroid taper. The child was given therapeutic anticoagulation with enoxaparin (day 3 of admission) and was given 5 mg/kg/day of aspirin (day 13 of admission). During the hospital stay, the gangrenous changes over the pinnae became clearly demarcated. Also, the patient developed new gangrenous changes over the toes (day 8 of admission) and fingers (day 11 of admission). He had a reduced volume in the left radial pulse and ulnar pulse when he developed gangrenous changes in his fingers. Doppler ultrasound showed partial thrombosis of the left proximal two thirds of the ulnar artery and distal one third of the radial artery. The rest of the limb vessels were normal. Because the patient had progressive skin lesions and very high levels of SARS-CoV-2 antibodies, with the possibility of direct organ injury resulting from the antibodies, a skin biopsy was tested and found to be positive for IgG antibodies to SARS-CoV-2. With a partial response to immune modulation, three cycles of plasmapheresis were done. Gradually, the skin lesions started to improve. There were clearly demarcated gangrenous changes of the bilateral pinnae, all toes of the right leg, four toes on the left leg except for the second toe, all fingers on the left hand, and all fingers on the right hand except for the ring finger (Figure 1C–F). A repeat antibody test to SARS-CoV-2 revealed a level of 12.5 U/mL. Repeat inflammatory markers improved (Table 1). Later, anticoagulation was switched to warfarin, with a target international normalized ratio of 2 to 3, and aspirin was continued. The patient was discharged with improving skin lesions after 19 days in the hospital (Figure 3). Two months after discharge, skin lesions over the face, upper limbs, lower limbs, and buttocks improved. The plan is to continue rehabilitation, and undergo cardiac surgery and cosmetic reconstruction at a later stage.

**Table 1:** Trends in laboratory parameters during the child’s hospital stay

Parameter	Normal value	DOA 1	DOA 4	DOA 8	DOA 13	DOA 19
Hb (g/dL)	11–14	10.6	7.0	11.9	10.9	10.9
Total leukocyte count (leukocytes/mm^3^)	5,000–15,000	34,600	10,300	15,200	12,910	13,880
Differential count: N, L, M, E	N, 37–60; L, 25–55; M, 2–8; E, 1–6	60, 30, 8, 2	65, 30, 3, 2	63, 30, 5, 2	70, 22, 7, 1	68, 27, 3, 2
Platelets (100,000/mm^3^)	2–5	0.91	2.7	3.1	4.8	5.4
ESR (mm/hour)	< 20	40	–	22	–	20
Prothrombin time (seconds)	11.1	14.2	16.8	–	14.6	31.0
INR	1.02	1.29	1.52	–	1.33	2.85
aPTT (seconds)	28.0	40.5	38.6	–	–	–
D-dimer (ng/mL)	< 500	2,232	1,509	–	–	532
C-reactive protein (mg/L)	0–5	175	95.4	16.9	32.9	12
Ferritin (ng/mL)	7–140	866	–	145	–	91.8
Urea (mg/dL)	11–38	79	21	–	–	–
Creatinine (mg/dL)	0.3–0.7	0.5	0.3	–	–	–
AST (U/L)	< 35	81	–	63	42	–
ALT (U/L)	< 35	36	–	62	34	–

ALT = alanine aminotransferase; aPTT = activated partial thromboplastin time; AST = aspartate aminotransferase; DOA = day of admission; E = eosinophils; ESR = erythrocyte sedimentation rate; Hb = hemoglobin; INR = international normalized ratio; L = lymphocytes; M = monocytes; N = neutrophils.

## DISCUSSION

COVID-19 infection has affected more than 40 million people worldwide and continues to cause infections with new variants. Children are affected less, with less severity.^1^ However, with reports of MIS-C, a greater number of children have required hospital admission, including stays in intensive care.^5^

Skin manifestations in COVID-19 infection are varied, with the incidence ranging from 0.2% to 20.4%,^2^ and include erythematous rash, urticaria, vesicles, pernio-like lesions, acral erythema, erythema multiforme, and chilblain-like lesions. In MIS-C, cutaneous manifestations have been reported in ∼ 80% of children.^3^^,^^4^ The most common finding is rash, which can be polymorphic, maculopapular, morbilliform, diffuse erythroderma, urticarial, or reticular. Erythema, edema, and induration of the extremities have also been reported frequently.^3^

Severe skin manifestations including acrocyanosis and gangrenous changes are reported less frequently with COVID-19 infection and MIS-C. Zhang et al.^6^ first reported acral ischemia in seven adults with COVID-19 infection. Subsequently, multiple cases of gangrenous changes with COVID-19 have been reported.^7^^–^^9^ These were attributed to the hypercoagulable state and disseminated intravascular coagulation induced by a cytokine storm in COVID-19 infection. Zhang et al.^10^ also reported the presence of antiphospholipid antibodies in patients with COVID-19. It has been observed that the presence of skin acrocyanosis and digital necrosis may suggest disease severity, as the thrombosis can be seen in multiple organs and may predict poor outcomes.^11^^,^^12^

To the best of our knowledge, only one report of two children with MIS-C with acral gangrene has been reported as of the time of this publication.^13^ Both of these children had hemodynamic instability, required intensive care, and developed gangrenous changes during the second week of illness. They improved with IVIG, steroids, and other supportive care. The main pathophysiology for the gangrenous changes in MIS-C is considered to be a result of endothelial dysfunction and microthrombi formation.

In our patient, there was retiform purpura with gangrenous changes of the ear during the first week of illness. He had very high levels of antibody titers to SARS-CoV-2, and features of both micro- and macroangiopathy. Although, he had multisystem involvement, there was no significant cardiac dysfunction and he did not require vasoactive support. Because inflammatory markers improved but the patient still had progressive skin lesions, we considered the possibility of direct organ injury resulting from circulating antibodies. A skin biopsy was positive for SARS-CoV-2 IgG antibodies. Although this may not confirm the pathophysiology, it provided some evidence of possible direct injury because of the high levels of circulating SARS-CoV-2 antibodies. After plasmapheresis, there were minimal new gangrenous lesions for 3 days. Gradually, the skin lesions began to improve and there were no new gangrenous changes. Whether the improvement in skin lesions and gangrenous areas was the result of immune modulation, plasmapheresis, or the natural course of the disease is difficult to interpret.

Children with TOF are at greater risk of thromboembolic complications. The various pathophysiological mechanisms include chronic hypoxemia leading to endothelial dysfunction that alters thrombomodulin levels, polycythemia, hyperviscosity, and associated iron deficiency.^14^^,^^15^ However, thromboembolic events causing gangrene in the extremities is seen less commonly. Animasahun and Amoah^16^ reported a 14-month-old female child with unoperated TOF with left forearm gangrene following streptococcal septicemia. Bing et al.^17^ reported a 4-year-old child with unoperated TOF with thrombosis of the lower abdominal aorta and gangrene of the right lower limb. We could not find any previous report of extensive gangrene similar to our case of unoperated TOF. In our patient, no evidence of thrombi or infective endocarditis was seen on echocardiography. He was at risk for thromboembolic complications resulting from the presence of chronic cyanosis, as evidenced by grade II clubbing and a baseline saturation of 84% to 86%. The occurrence of MIS-C in a child with TOF at risk for thromboembolic events might have triggered a cascade of events that led to such extensive gangrene. However, it is difficult to infer the major pathophysiological mechanism for the extensive gangrene in our patient.

Our patient had melena prior to admission, which can be a result of thrombocytopenia, stress-induced gastric ulcers, or gut involvement as a part of systemic hyperinflammation and angiopathy. There was no significant coagulopathy at admission. Children with TOF can have thromboembolic events in the gut that cause ischemia. The most common gastrointestinal complications reported are necrotizing enterocolitis and paralytic ileus, which are noted most commonly after surgical intervention. Spontaneous gut ischemia with or without acute illness is not seen often in TOF. We did not perform endoscopy to look for the cause of melena because there was no recurrence. It could have responded to antacids, improvements in platelet count, or immunomodulation.

To conclude, we report a case of unoperated TOF with MIS with retiform purpura with extensive gangrene involving the acral areas and ears in a young boy who improved, with residual deformity.
